# Near-Adult Heights and Adult Height Predictions Using Automated and Conventional Greulich–Pyle Bone Age Determinations in Children with Chronic Endocrine Diseases

**DOI:** 10.1007/s12098-021-04009-8

**Published:** 2022-02-01

**Authors:** Daniela Choukair, Annette Hückmann, Janna Mittnacht, Thomas Breil, Jens Peter Schenk, Abdulsattar Alrajab, Lorenz Uhlmann, Markus Bettendorf

**Affiliations:** 1 Division of Pediatric Endocrinology and Diabetology, University Children’s Hospital Heidelberg, Heidelberg, 69120 Germany; 2 Pediatric Radiology, University of Heidelberg, Heidelberg, Germany; 3 Institute of Medical Biometry and Informatics, University of Heidelberg, Heidelberg, Germany

**Keywords:** Automated bone age, Greulich–Pyle, Adult height predictions, Children with chronic endocrine diseases

## Abstract

**Objectives:**

To validate adult height predictions (BX) using automated and Greulich–Pyle bone age determinations in children with chronic endocrine diseases.

**Methods:**

Heights and near-adult heights were measured in 82 patients (48 females) with chronic endocrinopathies at the age of 10.45 ± 2.12 y and at time of transition to adult care (17.98 ± 3.02 y). Further, bone age (BA) was assessed using the conventional Greulich–Pyle (GP) method by three experts, and by BoneXpert™. PAH were calculated using conventional BP tables and BoneXpert™.

**Results:**

The conventional and the automated BA determinations revealed a mean difference of 0.25 ± 0.72 y (*p* = 0.0027). The automated PAH by BoneXpert™ were 156.26 ± 0.86 cm (SDS − 2.01 ± 1.07) in females and 171.75 ± 1.6 cm (SDS − 1.29 ± 1.06) in males, compared to 153.95 ± 1.12 cm (SDS − 2.56 ± 1.5) in females and 169.31 ± 1.6 cm (SDS − 1.66 ± 1.56) in males by conventional BP, respectively and in comparison to near-adult heights 156.38 ± 5.84 cm (SDS − 1.91 ± 1.15) in females and 168.94 ± 8.18 cm (SDS − 1.72 ± 1.22) in males, respectively.

**Conclusion:**

BA ratings and adult height predictions by BoneXpert™ in children with chronic endocrinopathies abolish rater-dependent variability and enhance reproducibility of estimates thereby refining care in growth disorders. Conventional methods may outperform automated analyses in specific cases.

## Introduction

Growth failure is very common in patients with chronic endocrinopathies, and the determination of skeletal maturity (bone age, BA) plays a pivotal role in endocrine diagnostic and is important to evaluate the individual growth potential [1, 2]. The most widely used methods for BA assessment are the Tanner–Whitehouse (TW2, TW3) methods [3, 4] and Greulich and Pyle (GP) method. For the latter, a trained rater with a background of pediatric endocrinology or pediatric radiology performs comparisons with the plates of the GP atlas from 1959 [4]. This manual procedure is time-consuming, requires experience, and is susceptible to considerable inter- and intrarater variability [2].

Recently, an automated image analysis method, BoneXpert™ has been introduced [5] and has been validated in patients with different endocrinopathies and with chronic renal failure [6–8]. BoneXpert™ uses an adopted adult height prediction method (BX), which is an improvement and extension of the Bayley–Pinneau method (BP) [9]. So far, it is validated for healthy children [10]. The objectives of this study were the validation of adult height predictions (BX) using automated Greulich–Pyle bone age determinations in children with chronic endocrine diseases**.** Therefore, these results were compared to adult height prediction calculations (BP), based on conventional assessments of GP performed by three independent experienced clinicians and to near-adult heights at the time of transition from pediatric to adult care.

## Material and Methods

At the University’s Hospital, 88 consecutive adolescents with chronic endocrine diseases were evaluated after achieving the primary pediatric treatment goals (girls: bone age ≥ 14 y and menarche; boys: bone age ≥ 16 y and voice brake) when > 98% of final adult height was reached and negligible growth potential was left [11]. To take part into this retrospective study written informed consent was obtained from each participant and their parents. The ethics committees of the University of Heidelberg approved the study which was performed according to the Declaration of Helsinki and conformed to legal and ethical norms (S-019/2011).

Six of the consecutive 88 patients were excluded as one radiograph of the left hand was unavailable and Digital Imaging and Communication in Medicine (DICOM) datasets from five patients were ineligible. Therefore, 82 radiographs of 48 females and 34 males were analyzed and corresponding heights were measured in an upright position, using a wall-mounted Harpenden stadiometer (Mentone Educational, Moorabbin, Victoria, Australia) at the chronological age of 10.45 $\pm$ 2.12 y and at time of transition (17.98 ± 3.02 y). Reference percentiles of Brandt and Reinken were used for calculation of standard deviation scores (SDS) [12].

Medications and information on potential osseous disorders were available. Patients with the following diagnoses were included: congenital adrenal hyperplasia (CAH), isolated growth hormone deficiency (iGHD), panhypopituitarism (MPHD), small for gestational age (SGA), and Ullrich–Turner syndrome (UTS). For the details of patients’ characteristics, see Table 1. All patients were treated according to international consensus guidelines [13–16].

**Table 1:** Patient characteristics at the time of transition from pediatric to adult care and heights (SDS) at mean chronological age of 10.5 ± 2.1 y

Diagnosis	Gender	Total	CAH	iGHD	MPHD	SGA	UTS
Gender (f/m) *n*		82 (48/34)	15 (7/8)	22 (9/13)	10 (5/5)	17 (9/8)	18 (18/-)
Chronological age (y)		17.98 ± 3.02	19.14 ± 2.63^#+^	17.45 ± 2.23^+#^	21.34 ± 4.67*^+&^	15.75 ± 1.31^#^*^&#^	18.18 ± 2.00^#+^
Near-adult height (cm)	female	156.38 ± 5.84	159.89 ± 3.82^+&^	159.56 ± 5.77^+^	160.03 ± 7.23^+&^	152.87 ± 4.44*^$^	154.13 ± 4.93*^$#^
	male	168.94 ± 8.18	173.33 ± 7.37^+$^	168.73 ± 6.42^+^*	170.36 ± 9.29^+&^	164.0 ± 4.75^+$#^	-
Near-adult heights SDS		**–**1.72 ± 1.2	**–**1.13 ± 0.96^+&^	**–**1.42 ± 1.03^&^	**–**1.25 ± 1.78^&^	**–**2.12 ± 0.89*	**–**2.51 ± 0.98*^§#^
Target height (cm)	female	161.87 ± 5.82	161.86 ± 5.46	162.69 ± 4.06	163.33 ± 6.54^+^	157.0 ± 7.13^#&^	163.46 ± 4.85^+^
	male	175.34 ± 6.42	176.88 ± 5.81	174.34 ± 5.54	176.6 ± 10.34	174.63 ± 6.31	-
Target height SDS		**–**0.88 ± 1.09	**–**0.72 ± 1.00	**–**0.95 ± 0.90	**–**0.42 ± 1.24	**–**1.41 ± 1.3	**–**0.70 ± 0.95
Differences Near-adult heights-SDS							
Target heights SDS		**–**0.84 ± 1.09	**–**0.41 ± 0.98^&^	**–**0.47 ± 0.79^&^	**–**0.83 ± 1.12^&^	**–**0.71 ± 1.16^&^	**–**1.81 ± 0.86*^§#+^

*CAH* Congenital adrenal hyperplasia*; f* Female; *iGHD* Isolated growth hormone deficiency; *m* Male; *MPHD* Panhypopituitarism; *SDS* Standard deviation score; *SGA* Small for gestational age; *UTS* Ullrich–Turner syndrome

Data are given as mean ± standard deviation (SD). Statistics by ANOVA: *p < 0.05 vs. CAH; ^§^*p* < 0.05 vs. GHD; ^#^*p* < 0.05 vs. MPHD; ^+^*p* < 0.05 vs. SGA; ^&^*p* < 0.05 vs. UTS

Two pediatric radiologists and one pediatric endocrinologist determined BA according to the atlas method of GP [4]. Since this method gives BA values at nonlinear intervals, BA is expressed as average of the younger and older BA respectively, when estimated BA was between the defined bone ages. For further statistical analysis, the mean of these three independent determinations of BA was used; named conventional BA and inter-rater variability was calculated. In addition, the automated assessment of BA was performed by BoneXpert™ version 2.1 (named automated BA, BoneXpert™, Visiana, Holte, Denmark, www.boneXpert.com). A more detailed description of this method is published elsewhere [17]. In brief, the automated BA consists of three layers of computation [17]. Layer A reconstructs and validates the borders of 15 bones: radius, ulna, all metacarpals, and the phalanges of finger I, III, V. The bone will be rejected if it is not placed correctly or it is severely dysmorphic. Layer B determines a so called intrinsic BA for each bone of the RUS bones (except metacarpal II and IV). Bones with BA deviated more than 2.4 y of the average BA were rejected. Average BA will not be calculated if less than 8 bones out of 13 were accepted. Layer C transforms the intrinsic BA into the scale of GP or TW. At the time of this study the BoneXpert™ covered the BA ranges 2.5–17 y for boys and 2–15 y for girls.

Height predictions were calculated using the conventional BA according to the methods of Bayley–Pinneau for girls and boys (conventional BP) [18] calculated by the anthropometric software GrowthXP from PC PAL, Stockholm, Sweden. Adult height predictions for girls and boys using the automated BA were calculated with BoneXpert™ (automated BX) according to Thodberg et al. [9] on www.bonexpert.com. Briefly, the growth potential (gp) was defined as: *gp* = *(H—h)/H* when H equals adult height and h represents actual height. The growth potential prediction is denoted *gp*_*pred*_ (BA, CA – BA), from this the so-called raw prediction of the adult height is derived: *H*_*raw*_ = *h/(1-gp*_*pred*_*).* The new method constructs *gp*_*pred*_* (BA, CA* – *BA)* as a nonlinear function of two variables. This is implemented as one neural network (a standard nonlinear regression method) for each gender as described in detail elsewhere [9]. In addition, target heights (cm; SDS) were calculated with the formula of Tanner (midparental height (+ 6.5 in boys or − 6.5 in girls), respectively) [19].

Data are given as mean ± standard deviation (SD), if not indicated otherwise. The differences between the conventional and automated BA were described using a Bland–Altman plot. The overall bias and limits of agreement are provided. Interrater variability expressed as Fleiss´κ was also calculated. All data were assessed for normal distribution by the Kolmogorov–Smirnov test. Correlation coefficients were calculated according to Pearson. A *p* value < 0.05 was considered as statistically significant. Since this was an exploratory analysis, no adjustment for multiple testing was done. Stat View version 5.0 1998 and the software R in combination with the package psychometric were used for statistical analysis [20, 21].

## Results

None of the 82 analyzed images were rejected by BoneXpert™. The inter-rater variability of the conventional BA determination according to Greulich and Pyle performed by three experienced raters was 0.88.

**Fig. 1:**
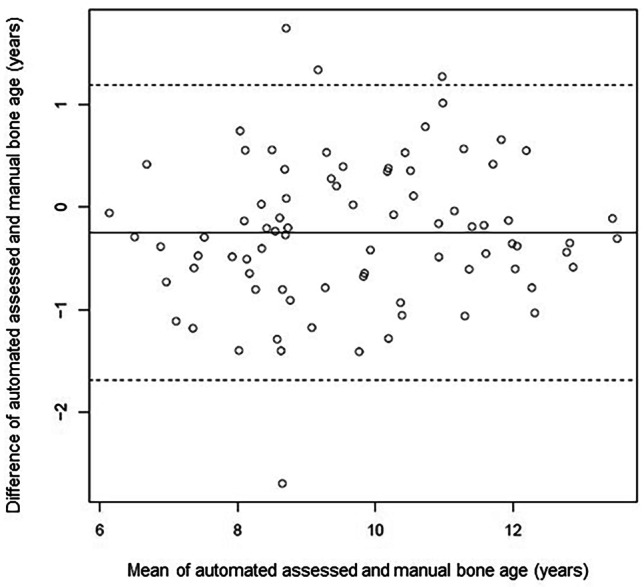
Bland–Altman plot demonstrates the relationship between automated and conventional bone age ratings (mean of three independent ratings). The differences between the two methods are depicted against the mean of these two methods. The dashed lines indicate 2 SD

There was a good correlation between the conventional and the automated bone age ratings (*r* = 0.843; *p* < 0.001). The automated and the conventional BA determinations differed by − 0.25 ± 0.72 y (*p* = 0.0027) (Table 2) ranging from − 1.67 to 1.19 y. The conventional method tended to rate bone ages slightly more mature. The exact distribution is shown in Fig. 1 using a Bland–Altman plot. Further, the mean differences between the automated and conventional method for female and male patients were significantly different (females, − 0.16 ± 0.68 y; males, − 0.37 ± 0.77 y; *p* = 0.001).

**Table 2:** Comparison of conventional bone age determination according to Greulich and Pyle (conventional BA) and automated bone age determination (automated BA)

Diagnosis	Total	CAH	iGHD	MPHD	SGA	UTS
Chron. age (CA; y)	10.45 ± 2.12	8.75 ± 1.60^§#&^	11.16 ± 1.85*^+^	12.12 ± 2.02*^+^	9.66 ± 1.53^§#^	10.82 ± 2.22*
Automated BA (y)	9.59 ± 1.86	10.78 ± 1.85^+^	9.63 ± 2.08^+^	9.60 ± 1.6	8.43 ± 1.45*^§&^	9.67 ± 1.51^+^
Differences Automated BA – CA (y)	–0.86 ± 2.26	2.04 ± 2.67^§#+&^	–1.53 ± 1.67*	–2.52 ± 2.01*	–1.23 ± 1.05*	–1.15 ± 1.5*
Conventional BA (y)	9.84 ± 1.78	11.06 ± 1.79^#+&^	9.94 ± 1.99	9.80 ± 1.57*	8.98 ± 1.43*	9.66 ± 1.52*
Differences Conventional BA – CA (y)	–0.61 ± 2.20	2.32 ± 2.57^§#+&^	–1.22 ± 1.55*	–2.32 ± 1.8*^+^	–0.68 ± 0.97*^#^	–1.16 ± 1.46*
Differences Automated BA – Conventional BA (y)	–0.25 ± 0.72	–0.28 ± 0.69	–0.31 ± 0.57	–0.2 ± 0.97	–0.54 ± 0.77^&^	0.01 ± 0.68

*BA* Bone age; *CA* Chronological age; *CAH* Congenital adrenal hyperplasia*; f* Female; *iGHD* Isolated growth hormone deficiency; *m* Male; *MPHD* Panhypopituitarism; *SGA* Small for gestational age; *UTS* Ullrich–Turner syndrome

Data are given as mean ± standard deviation (SD). Statistics by ANOVA: *p < 0.05 vs. CAH; ^§^*p* < 0.05 vs. GHD; ^#^*p* < 0.05 vs. MPHD; ^+^*p* < 0.05 vs. SGA; ^&^*p* < 0.05 vs. UTS

Overall the automated BA was retarded by 0.86 ± 2.26 y (Table 2). The respective bone ages were accelerated by 2.04 y in CAH, but retarded in MPHD by − 2.52 y, and moderately retarded in SGA, UTS, and iGHD (Table 2). Conventional and automated bone age determinations were similar in all patient groups (*p* = ns), except in patients with SGA (*p* < 0.05) (Table 2).

The mean near-adult heights in females and in males [females 156.38 ± 5.84 cm (SDS − 1.91 ± 1.15), males 168.94 ± 8.18 cm (SDS − 1.72 ± 1.22)] were significantly lower than target heights in females [161.87 ± 5.82 cm (SDS − 1.00 ± 1.14)] and in males [175.34 ± 6.42 cm (SDS − 0.71 ± 1.00)], respectively (*p* < 0.001) (Table 1).

**Fig. 2:**
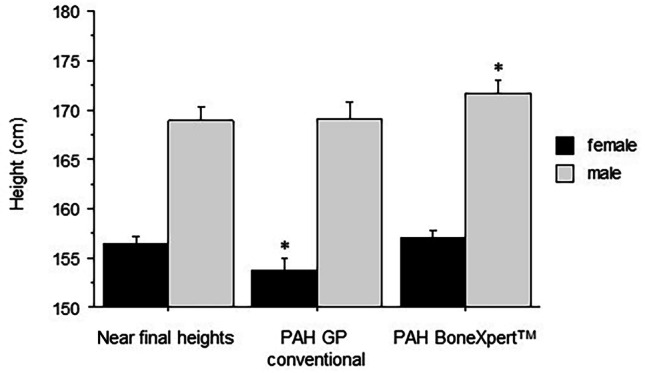
Near-adult heights (cm) and adult height predictions (cm) using the tables of Bayley– Pinneau and conventionally determined bone age according to Greulich and Pyle (conventional BP), and by BoneXpert™ (automated BX), respectively are depicted. Mean ± SD, *indicating *p* < 0.05. (SDS—Near-final heights: Females: –1.91 ± 1.15; Males: –1.72 ± 1.22; PAH GP conventional Females: –2.56 ± 1.5; Males: –1.66 ± 1.56; PAH BoneXpert ™ Females: –2.01 ± 1.07; Males: –1.29 ± 1.06)

The predicted adult heights using the method of BoneXpert™ (automated BX) were 156.26 ± 0.86 cm (SDS − 2.01 ± 1.07) in girls matching near-adult heights (Δ 0.58 ± 0.84 cm; *p* = ns) (Fig. 2). In contrast, the predicted adult heights using conventional BP were 153.95 ± 1.12 cm (SDS − 2.56 ± 1.5), in girls underestimating near-adult heights by 2.43 ± 0.84 cm (*p* < 0.01) (Fig. 2). The automated BX were 171.75 ± 1.6 cm (SDS − 1.29 ± 1.06), in boys, overestimating near-adult heights by 2.81 ± 2 cm (*p* < 0.05) (Fig. 2). Conventional BP in boys corresponded to near-adult heights in boys [169.31 ± 1.6 cm (SDS − 1.66 ± 1.56)]; Δ 0.37 ± 1.4 cm; *p* = ns) (Fig. 2).

Conventional BP and automated BX were analyzed separately in CAH and MPHD because of distinct differences in bone ages and chronological ages (CAH: 2.04 ± 2.67 y; MPHD: − 2.52 ± 2.01 y, respectively). Predicted adult heights using both methods were similar as near-adult heights in females regardless of bone age acceleration and retardation (Table 3). Automated BX overestimated significantly predicted adult heights in males with MPHD when bone ages were retarded (9.3 ± 3.02 cm; *p* < 0.05) (Table 3).

**Table 3:** Predicted adult heights using the method of Bayley–Pinneau (conventional BP) and BoneXpert™ (automated BX) in patients with accelerated bone age (BA) [congenital adrenal hyperplasia (CAH)] and delayed BA [panhypopituitarism (MPHD)]

		CAH (cm)	CAH (SDS)	MPHD (cm)	MPHD (SDS)
Female	Near-adult heights	160.0 ± 3.8	–1.37 ± 0.75	161.5 ± 7.1	–1.08 ± 1.42
	Conventional BP	160.0 ± 8.3	–1.37 ± 1.64	162.5 ± 6.6	–0.88 ± 1.33
	Automated BX	162.2 ± 6.0	–0.94 ± 1.17	162.6 ± 2.8	–0.86 ± 1.05
Male	Near-adult heights	173.3 ± 7.4	–1.03 ± 1.13	170.4 ± 6.4	–1.48 ± 2.26
	Conventional BP	170.0 ± 9.6	–1.55 ± 1.61	173.6 ± 7.9	–0.98 ± 2.76
	Automated BX	177.5 ± 6.5	–0.38 ± 1.02	179.7 ± 4.9*	–0.33 ± 0.77

*CAH* Congenital adrenal hyperplasia; *MPHD* Panhypopituitarism

The difference of conventional BA and chronological age (CA) in patients with CAH was 2.32 ± 2.57 y and was 2.04 ± 2.67 y when BA was estimated by BoneXpert™ (automated BA). The difference of conventional BA and CA in patients with MPHD was –2.32 ± 1.8 y and was –2.52 ± 2.01 y when automated BA was utilized. Data are given as mean ± standard deviation (SD). Statistics by *t*-test: *p < 0.05 vs. near adult height

## Discussion

As far as the authors know, this study represents the first comparison of observed near-final heights and the recent adult height calculation method based on automated bone age determination (BoneXpert™) with the conventional PAH method by Bayley − Pinneau based on bone age determination according to Greulich and Pyle in children with various chronic endocrinopathies. In general, there was a good agreement of automated BX in girls, regardless of bone age deviations from chronological age. The recent method of automate BX implements a nonlinear growth potential function [9]. This is more graduated and therefore more precise than the conventional BP using the tables of Bayley − Pinneau which rates only for three ranges namely advanced (BA > CA > 1 y), normal BA (CA = BA ± 1 y) and retarded (BA < CA <  − 1 y). The present results applying automated BX in girls are in accordance with the results of Unrath et al. [10] and Thodberg et al. [22], who investigated healthy children with short stature. Martin et al. reported a slight underprediction of automated BX in girls with short stature by 0.8 cm when bone age was younger than 12 y [23]. This observation was explained by an individual, unpredictable growth pattern of six included girls.

Remarkably, automated BX overestimated adult heights in boys, especially, when bone ages were severely retarded. This observation was confirmed by Thodberg et al. [22]. In their study, height predictions in boys using automated BX did not outperform the conventional method in boys indicating an inherent weak spot. It is known, that conventional BP systematically overestimates adult height in boys, especially when bone age is retarded [24] or in constitutional tall stature [25].

These observations cannot be explained by a systematic error in bone age determination of automated BA, because the present study and all other studies reported a good accordance with the conventional BA assessment (Fig. 1 and Table 1) [10, 17]. This difference is similar to the deviation between two manual raters [17]. Further, this remarked effect is not caused by incorrect bone age determination due to dysmorphic bones, because none of the images were rejected by BoneXpert™.

The residual errors of growth prediction in both methods arise from various sources: (a) conventional growth prediction models presumed a linear dependence between adult height and BA, but puberty and its growth spurt are dynamic processes and not concordant with bone age advancement. Each child experiences an individual growth pattern. The tables of Bayley and Pinneau were initially evaluated in healthy children but thereafter adapted as benchmark to children with various growth disorders and treatments. Implementation in clinical routine of growth disorders revealed a valuable tool in treatment control and expectations [26], (b) incorrect measurements of height and (near-) adult heights, (c) unpredictable influences on growth and pubertal development, such as nutrition, genetic, and environmental factors. But a clear advantage using automated BX is that the bone age determination is not impaired by rater variability and can be therefore easily used for clinical studies. Automated bone age determination by BoneXpert™ can be used as a reliable tool and efficient method because it is time- and cost-saving. It is reliable also when bone age is accelerated or retarded [6, 7, 10]. Apart from children with chronic kidney disease, in which automated BA tended to underestimate acceleration or retardation of bone age [8], this difference can be probably explained by the renal osteodystrophy in this cohort.

For evaluation of bone morphology, a rating of the radiographs by an expert remains mandatory and cannot be replaced by computerization. A limitation of this study is the small numbers of patients in each group. Therefore, future multicenter studies with a larger sample size are needed to evaluate accuracy of automated BX.

Taking the described limitations into account, the new prediction method is superior to the conventional BP in girls, regardless of bone age deviation from chronological age. Automated BX tends to overestimate PAH in boys, especially, when bone age is retarded.

## Conclusion

Bone age determination is a standard investigation in the workup of growth disturbances in children with various pediatric diseases [27], but also in orthodontics and pediatric orthopedics [28]. Further, it is used for legal issues, especially to determine a person's age, based on skeletal radiographs. However, according to the statement of the European Society of Pediatric Radiology musculoskeletal task force group, exact determination of chronological age of a person cannot be done with sufficient accuracy with existing methods [29]. Based on the bone age determination, several prediction models for adult heights were established. The accuracy of the predicted adult heights is limited due to the growth pattern that can be influenced by medication, nutrition, individual variation in pubertal height gain, and environmental factors [30]. This biological variation is not possible to overcome with mathematical prediction models. Therefore, the accuracy of bone age assessment should be optimized to improve the accuracy of height prediction methods. Using automated bone age determination an inter- and intrarater error is eliminated, and consecutively, reproducibility of adult height prediction improves.

## Declarations

### Code Availability

Stat View version 5.0 1998.
